# Internalized homonegativity moderates the association between attachment avoidance and emotional intimacy among same-sex male couples

**DOI:** 10.3389/fpsyg.2023.1148005

**Published:** 2023-03-29

**Authors:** Mónica Guzmán-González, Fabiola Gómez, Joaquín Bahamondes, Jaime Barrientos, Lusmenia Garrido-Rojas, Ricardo Espinoza-Tapia, Giulia Casu

**Affiliations:** ^1^Laboratory of Research in Attachment, Couple Relationships and Family, School of Psychology, Universidad Católica del Norte, Antofagasta, Chile; ^2^School of Psychology, Pontificia Universidad Católica, Santiago, Chile; ^3^School of Psychology, Universidad Católica del Norte, Antofagasta, Chile; ^4^Faculty of Psychology, Universidad Alberto Hurtado, Santiago, Chile; ^5^Department of Psychology, Universidad Católica del Maule, Talca, Chile; ^6^Department of Psychology, University of Bologna, Bologna, Italy

**Keywords:** attachment anxiety, attachment avoidance, emotional intimacy, internalized homonegativity, same-sex male couples

## Abstract

**Introduction:**

The present study aimed to examine dyadic associations between attachment insecurity and emotional intimacy in same-sex male couples, and to investigate whether and how each partner’s internalized homonegativity (IH) moderated these associations.

**Methods:**

The sample included 138 same-sex male couples. Both dyad members completed self-report measures of attachment insecurity, emotional intimacy, and IH. The actor-partner interdependence model with moderation analysis was applied.

**Results:**

Indicated that higher levels of actor’s and partner’s attachment anxiety and attachment avoidance were associated with lower actor’s emotional intimacy. IH moderated the partner effects of attachment avoidance on emotional intimacy. The partner’s higher attachment avoidance was associated with one’s own lower emotional intimacy at low (but not high) levels of one’s own IH and at high (but not low) levels of the partner’s IH.

**Discussion:**

Findings suggest that the partner’s attachment avoidance may differently affect one’s own emotional intimacy depending on the IH levels of both dyad members. Helping partnered sexual minority men decrease attachment insecurity while recognizing their own and their partners’ IH may promote relationship quality.

## 1. Introduction

Same-sex male couples form and maintain their relationships in diverse and progressively changing socio-cultural contexts (Rostosky and Riggle, 2017). Although they have achieved more rights in recent decades, including the legal recognition of their unions, they are embedded in a culture that still privileges heterosexual relationships (ILGA-Europe, 2023). Noteworthy, sexual prejudice and stereotyping and discrimination against LGBT individuals are still widespread even in most modern Western societies (Salvati et al., 2020).

Same-sex male couples face particular challenges related to the marginalized status of their relationships (Meyer, 2003; Pepping et al., 2018), a contextual element that is important to consider when studying relationship quality in this group (Rostosky and Riggle, 2017). Indeed, within the framework of minority stress theory (Meyer, 2003), the stigma that comes from being part of a sexual minority increases the risk of experiencing negative individual and relational outcomes among LGBT people (Meyer, 2003, 2015; Newcomb and Mustanski, 2010). In particular, the internalization of societal stigma and negative attitudes toward sexual minority individuals, a phenomenon referred to as internalized homonegativity (IH; Herek et al., 2009), has negative effects on couple relationship quality (Frost and Meyer, 2009; Cao et al., 2017; Feinstein et al., 2018; Pepping et al., 2018; Gonçalves et al., 2020). Although these effects are experienced by all sexual minority individuals in the LGBT community, studies indicate that gay and bisexual men are targets of more discrimination and hostile heterosexist attitudes than lesbian and bisexual women (Nierman et al., 2007; Barrientos and Cárdenas, 2013; Frost et al., 2016; Tsai et al., 2021), whereas no data are currently available for other sexual minority groups. Accordingly, there is evidence that sexual minority men internalize homonegativity at higher rates and experience more negative effects on their mental health because of a greater pressure to conform to heteronormative gender roles and the internalization of sexual prejudice (Bahamondes, 2016; Feinstein and Dyar, 2017; de Graaf and Picavet, 2018; Lee et al., 2022).

Within the theoretical formulations applied to understand couple relationship dynamics, attachment theory (Bowlby, 1979, 1980) plays a preponderant role. In this context, there is ample evidence that the degree of attachment insecurity is associated with different aspects of relationship quality (Li and Chan, 2012; Feeney, 2016; Mikulincer and Shaver, 2016), including emotional intimacy (Gabbay and Lafontaine, 2020). However, most studies have addressed the association between attachment and intimacy in different-sex couples, and we are not aware of studies in same-sex male couples.

Moreover, few studies have integrated these two widely supported perspectives (minority stress theory and attachment theory) regarding the impact of IH and attachment insecurity on intimacy of same-sex male couples. Such a gap in the literature would be explained by the notion that attachment processes unfold uniformly, regardless of sexual orientation.

Another gap in research on same-sex couple functioning is that studies have tended to privilege an individual over a dyadic approach, as highlighted in reviews on the effects of minority stressors (Rostosky and Riggle, 2017). The present study addresses these oversights by exploring the association between attachment insecurity and a key aspect of couple functioning, namely, emotional intimacy, using a dyadic approach where both partners’ perspectives are considered, and if this association is moderated by IH.

The relevance of adopting a dyadic perspective in the study of couple relationships lies in the possibility of capturing the mutual influence between partners. Couple relationships are dynamic and reciprocal, as the attitudes, emotions, and behaviors of one partner influence and are influenced by those of the other partner (Mikulincer and Shaver, 2016). A dyadic modeling approach that allows to capture the interconnectedness and interdependencies in couples is the actor-partner interdependence model (APIM) proposed by Kenny et al. (2006). The APIM uses the couple as the unit of analysis and allows to simultaneously estimate actor and partner associations. Individual, within-partner associations between actors’ predictors and their own outcome variables are referred to as actor effects, and cross-partner associations between partners’ predictors and actors’ outcomes are referred to as partner effects (Kenny et al., 2006).

### 1.1. Adult attachment theory as a conceptual framework for understanding emotional intimacy

Emotional intimacy is a relational process inherent to close relationships, defined by Sinclair and Dowdy (2005) as the perception of closeness that allows sharing of personal feelings, accompanied by expectations of understanding, affirmation, and demonstrations of caring. Emotional intimacy is a powerful predictor of psychological and physical well-being (Hook et al., 2003; Stadler et al., 2012), as well as of relationship satisfaction (Greeff and Malherbe, 2001; Laurenceau et al., 2005; Guschlbauer et al., 2019; Štulhofer et al., 2020; Guzmán-González et al., 2021).

Attachment theory, formulated by Bowlby (1979, 1980), is a privileged conceptual framework for understanding how people experience emotional intimacy in couple relationships. Hazan and Shaver (1987) were pioneers in this field by proposing the existence of a parallel between the infant-caregiver bond and romantic love, arguing that the need for comfort and security remains in adulthood, but is sought primarily in the partner rather than in the parents.

Attachment theory posits that early repeated experiences with significant others are internalized in a set of beliefs about self and others, called internal working models, which guide social interactions, especially in close relationships (Mikulincer and Shaver, 2016). These individual representations explain, at least in part, how partners behave with each other in their interactions and build their relational intimacy (Constant et al., 2021). From this perspective, a widely accepted notion is that romantic attachment can be described along two dimensions: attachment anxiety and attachment avoidance, which are associated with the model of self and others, respectively (Brennan et al., 1998; Mikulincer and Shaver, 2016). Attachment anxiety refers to the fear of abandonment in relationships and is based on a negative view of the self. People with high anxiety manifest an exaggerated need for approval, an exacerbation of protest reactions, and a constant search for emotional reassurance and closeness. Attachment avoidance refers to discomfort with closeness and dependence, reluctance to seek support, and a tendency to deactivate emotional needs, based on expectations of rejection due to a negative model of others (Shaver and Mikulincer, 2002; Mikulincer et al., 2003).

To understand emotional intimacy within this framework, attachment theorists propose that more securely attached individuals, who have positive models of self and others, feel comfortable with intimacy and closeness. Instead, people who are more anxiously or avoidantly attached experience more difficulties in negotiating issues related to closeness and distance (Pistole, 1994; Mikulincer and Shaver, 2016). Individuals with higher attachment anxiety experience unmet needs for love and closeness that make them more likely to demand intimacy in ways that can be intrusive, paradoxically facilitating distance or withdraw responses (Feeney and Noller, 1991; Bradford et al., 2002), and they are more prone to sharing personal information in a non-constructive way (Bradford et al., 2002). Conversely, individuals with higher attachment avoidance tend to keep emotional distance from their partners and to exacerbate their independency, thereby reducing their own intimacy-promoting behavior and their responsiveness to the partner’s intimacy needs. Moreover, their discomfort with intimacy makes them reluctant to disclosure of personal feelings (Shaver and Mikulincer, 2002; Mikulincer et al., 2003; Mikulincer and Shaver, 2016).

Accordingly, studies in this field reveal that the degree of attachment insecurity has a role in perceived emotional intimacy (Li and Chan, 2012; Feeney, 2016; Mikulincer and Shaver, 2016). Cross-sectional and longitudinal studies of individuals and couples in heterosexual relationships reported that higher attachment anxiety and attachment avoidance were associated with lower intimacy (Collins et al., 2002; Collins and Feeney, 2004; Pielage et al., 2005; Rholes et al., 2011; Dandurand and Lafontaine, 2013; Constant et al., 2021). A recent dyadic study of different-sex couples reported a negative actor effect of attachment anxiety on perception of intimacy, whereas actor’s and partner’s attachment avoidance were both related to actor’s lower perceived intimacy (Gagné et al., 2021).

We are not aware of any studies exploring emotional intimacy in same-sex male couples, but an individual-based study about sexual intimacy in the context of same-sex relationships arrived at a similar conclusion that attachment insecurities are linked to lower sexual intimacy (Gabbay and Lafontaine, 2020). It is worth noting that a more consistent and strong association has been detected for attachment avoidance than for attachment anxiety (Pielage et al., 2005; Constant et al., 2021; Gagné et al., 2021).

### 1.2. The moderating role of IH in the association between attachment insecurity and emotional intimacy

The studies mentioned above provide support for the notion that actor’s and partner’s attachment insecurities are linked to one crucial aspect of relationship functioning such as emotional intimacy. However, an important question that has not been addressed yet is whether a minority stressor like IH represents a risk factor that might increase the strength of the dyadic associations between attachment and emotional intimacy in same-sex male couples.

Minority stress theory posits that being part of stigmatized minority groups is a source of stress that produces negative effects on individual and relational well-being (Meyer, 2003). Four minority stressors have been identified which are placed on a continuum from distal (i.e., external) to proximal (i.e., psychological): distal stressors include acute and chronic forms of discrimination and victimization and everyday discrimination (e.g., microaggressions); proximal stressors include expectations of rejection and discrimination (i.e., felt stigma); stigma concealment; and internalized homonegativity (Frost et al., 2022).

IH is manifested through negative attitudes and beliefs toward LGBT people, feelings of shame and rejection toward one’s sexual orientation, concealment of interaction with other LGBT people, fear of public identification (Meyer, 2003; Tozer and Hayes, 2004; Berg et al., 2013), and more or less conscious negative appraisals of same-sex relationships (Lingiardi et al., 2012). IH has become a focus of research interest because it is argued that a large proportion of LGBT people experience at least some degree of IH, which increases the risk of experiencing mental health problems (Frost and Meyer, 2009; Szymanski and Ikizler, 2013; Denton et al., 2014). IH is also a predictor of lower relationship quality, and there is evidence that stressors of this type, which are more chronic and subtler than explicit events such as victimization episodes, are more likely to impair the quality of couple relationships (Randall and Bodenmann, 2009; Feinstein et al., 2018). Individuals with high IH are caught in the ambivalence of yearning and needing a partner relationship that goes against their beliefs or values, which can translate into shame about publicly exposing the relationship, less supportive and emotionally responsive behaviors in their couple relationships, as well as lower levels of intimacy (Mohr and Jackson, 2016). Noteworthy, there is evidence that discomfort with same-sex sexual intimacy is linked to the endorsement of sexist social attitudes, suggesting that the adoption of sexist standards may be associated with the belief that correct sexuality embraces roles and morality coherent with the normative heterosexual model (López-Sáez et al., 2020).

Consistent with this theoretical link, studies of LGBT individuals show that IH is negatively associated with relationship quality (Frost and Meyer, 2009; Calvillo et al., 2018; Pepping et al., 2018), including lower levels of closeness and emotional intimacy (Mohr and Daly, 2008; Szymanski and Hilton, 2013; Guschlbauer et al., 2019). Studies of dyads, rather than individuals, are still scarce in the context of same-sex relationships and focused on other aspects of relationship quality. However, their findings are consistent in suggesting a detrimental effect of IH for couples’ functioning (Feinstein et al., 2018; Totenhagen et al., 2018; Li et al., 2022). Totenhagen et al. (2018) found that among same-sex couples, levels of actor’s IH interacted with actor’s daily stress levels, such that only individuals high in IH reported lower relationship quality on days of higher perceived stress. In another study of young same-sex male couples (Feinstein et al., 2018), higher actor’s levels of minority stress were associated with lower actor’s relationship quality, and higher levels of both actor’s and partner’s internalized stigma were linked to more actor’s reported negative interactions. A more recent study of same-sex couples reported that higher levels of actor’s IH were related to a higher probability of partner’s psychological violence perpetration when actor’s levels of commitment were low (Li et al., 2022). Hence, these dyadic studies suggest actor- and partner-level influences of IH on relationship functioning. Despite these advances, important gaps still exist.

Even though the impact of IH has been explored on different aspects of relationship functioning, its moderating role on the relationship between attachment and emotional intimacy from a dyadic perspective remains unclear. Karney and Bradbury (1995) vulnerability-stress-adaptation model posits that individual vulnerability factors (such as attachment insecurity) can especially impair relationship quality if combined with stressors (such as IH). Therefore, it offers theoretical support for a possible moderating role of IH in the link between attachment and emotional intimacy. Indeed, IH involves the materialization of most proximal minority stress processes, as it entails the internalization and application to the self of heterosexist and heteronormative societal attitudes (Frost and Meyer, 2009) which lead to negative self-appraisals and intrapsychic conflict (Herek, 2004). This may influence the activation of the attachment system, which aims to ensure safety in times when challenges to one’s sense of well-being are most prominent (Bowlby, 1980).

Considering that attachment insecurity is particularly activated under situations of threat or stress (Feeney, 2016), it is likely that attachment insecurities have a more negative impact on emotional intimacy for couples where their members manifest higher levels of IH. Specifically, having higher levels of IH would intensify the detrimental effect of holding a negative view of self (one core aspect of attachment anxiety) on emotional intimacy through hyperactivating strategies, for example by favoring a focus on reducing fear of rejection rather than on sharing reciprocal intimacy and enjoyment (Gillath et al., 2016; Mikulincer and Shaver, 2016). A higher IH might also increase the negative effect of a negative view of others (a core aspect of attachment avoidance) on emotional intimacy through deactivating strategies such as emphasizing the need to place limits on closeness based on distrust in others. Similarly, having a partner who holds feelings of shame and rejection toward his sexual orientation might increase the negative effects of the partner’s attachment anxiety and avoidance on one’s own emotional intimacy. Indeed, this combination of factors (high IH with high attachment anxiety or high attachment avoidance) in one partner may favor defensive processes that interfere with the perception of responsiveness in the other partner, reducing his sense of intimacy and shared emotions (Mohr and Jackson, 2016).

### 1.3. The present study

The present study aimed to examine actor and partner associations between attachment insecurity and emotional intimacy in same-sex male couples, and to analyze whether IH moderated these dyadic associations.

With these objectives, we may contribute to expand previous research in several ways. To our knowledge, no study has explored associations between attachment insecurities and emotional intimacy in same-sex male couples. Second, this is the first study to explore the moderating role of a proximal minority stressor on the relationship between romantic attachment and a core aspect of relationship functioning like intimacy from a dyadic perspective. Third, our study integrates two sounded theoretical perspectives: attachment and minority stress theory. Most importantly, this study has potential relevance for theory and practice. At the theoretical level, it may clarify whether, among same-sex couples, attachment insecurity plays the same effects on aspects of relationship functioning as in different-sex couples, and provide preliminary evidence of the role of IH within the framework of attachment theory. Furthermore, elucidating the dyadic interactive effects of romantic attachment and IH on emotional intimacy among same-sex male couples might offer valuable insights for more-culturally competent, couple-based psychotherapeutic, and counseling practice (Scott et al., 2019).

Based on previous evidence and theoretical considerations, we hypothesized that actor’s and partner’s attachment anxiety and attachment avoidance would be negatively associated with actor’s emotional intimacy. As for the moderating role of IH, we hypothesized that higher levels of IH, which constitutes a stressor that interacts with attachment insecurities facilitating the individual’s deployment of the secondary strategies of the attachment system (hyperactivation or deactivation) (Karney and Bradbury, 1995; Feeney, 2016), will exacerbate the posited dyadic associations of attachment insecurities with lower emotional intimacy. Specifically, we hypothesized that one’s own IH will moderate the actor effects of attachment insecurities on emotional intimacy, such that higher actor’s IH would intensify the negative effects of both actor’s attachment anxiety and actor’s attachment avoidance on actor’s emotional intimacy. Similarly, we hypothesized that partner’s IH will moderate the partner effects of attachment insecurities on emotional intimacy, such that higher partner’s IH would intensify the negative effects of both partner’s attachment anxiety and partner’s attachment avoidance on actor’s emotional intimacy. Due to the paucity of previous research, we did not formulate hypotheses on the moderating role of partner’s IH on actor-level associations nor on the moderating role of actor’s IH on partner-level associations, which were analyzed in an exploratory way.

The above associations were tested using the actor-partner interdependence moderation model (APIMoM; Garcia et al., 2015), an extension of the APIM that incorporates moderation.

## 2. Materials and methods

### 2.1. Participants’ characteristics

The sample included 138 same-sex male couples from Chile. The 276 partners were aged 18 to 76 years (*M* = 32.75, *SD* = 9.89), 61.6% (*n* = 170) had higher (technical or university) education, and 71.4% (*n* = 197) were employed. For the 138 couples, relationship length ranged from 6 months to 36 years (*M* = 5.05 years, *SD* = 5.99), and 70.3% of couples (*n* = 97) had been together for 1 to 4 years. Most couples (62.3%, *n* = 86) were cohabiting, and 15.1% of these (*n* = 13) were in a civil union. In 8% of couples (*n* = 11) one or both partners had children, in 76.1% (*n* = 105) one or both partners were highly educated, and in 87.7% (*n* = 121) one or both partners were employed.

### 2.2. Procedure

Data for the present study came from a larger project examining relationship quality in same-sex couples and were approved by the University Ethics Board. The recruitment process was carried out through a non-probabilistic sampling by quotas according to age and gender identity. Sample size was established with an *a priori* power analysis conducted with G*Power 3.1 (Faul et al., 2007), following sample size recommendations in multiple regression analysis (Kenny and Cook, 1999). The power analysis indicated that a minimum of 134 couples would be needed to detect small-to-medium-sized effects (*f^2^* = 0.12) with a power of 80% and an alpha of 0.05 for a multiple linear regression with eight predictors (four main effects and four interaction effects).

To take part in the study, partners had to be 18 years or older, be involved in a same-sex male couple relationship for at least 6 months, and both partners had to be willing to participate. Data collection was carried out through the SurveyMonkey platform. A team of research assistants from the main regions of Chile were in charge of recruiting potential couples *via* advertisements on social networks, dissemination in organizations of sexual diversity, personal contacts, and the snowball technique. If both members of a couple agreed to participate, the research assistant provided them a link to the online survey along with an ID code to match partners’ responses. Participants were asked to independently enter the ID, read the instructions, declare their eligibility criteria (otherwise, they were not able to continue the survey), sign the online consent form, and complete a series of questionnaires. They were instructed to answer the survey individually, and to not discuss the questions or answers with their partner. Upon completion, participants received a compensation for the time spent completing the survey, consisting in $25 USD. All research assistants were required to sign a confidentiality statement.

### 2.3. Measures

#### 2.3.1. Sociodemographic information

Participants responded to a sociodemographic form asking for age, educational level, job status, length of relationship, and union (being in a civil union or not), cohabitation (cohabiting with the partner or not), and parental status (having children or not).

#### 2.3.2. Romantic attachment

Attachment insecurity was evaluated with the Experiences in Close Relationship questionnaire (ECR, Brennan et al., 1998) in its Chilean validated 12-item version (Guzmán-González et al., 2020a). The ECR measures adult attachment on two dimensions: attachment anxiety (e.g., I worry that romantic partners will not care about me as much as I care about them; I worry a fair amount about losing my partner) and attachment avoidance (e.g., I do not feel comfortable opening up to romantic partners; Just when my partner starts to get close to me I find myself pulling away). Each item is rated on a 7-point scale from 1 (disagree strongly) to 7 (agree strongly). Higher scores indicate higher levels of attachment insecurity. In Chilean samples, Cronbach’s alpha coefficient ranged from 0.72 to 0.83 for the anxiety subscale and from 0.78 to 0.89 for the avoidance subscale (Guzmán-González et al., 2020a). Reliability in the present sample was Cronbach’s α = 0.81 and 0.77 for attachment anxiety and attachment avoidance, respectively.

#### 2.3.3. Emotional intimacy

It was assessed using the Emotional Intimacy Scale (EIS; Sinclair and Dowdy, 2005), in its Chilean validated version (Guzmán-González et al., 2021). This 5-item self-report scale measures perceptions of being validated (e.g., My partner completely accepts me as I am), understood (e.g., My thoughts and feelings are understood and affirmed by my partner), and cared for (e.g., My partner cares deeply for me). Items are rated on a 5-point scale from 1 (strongly disagree) to 5 (strongly agree), with higher scores reflecting greater emotional intimacy. The scale showed good reliability, with Cronbach’s *α* coefficient of 0.88 and 0.90 for the original and Chilean validated version, respectively. Substantial evidence has been provided for construct and criterion-related validity of the EIS (Sinclair and Dowdy, 2005; Guzmán-González et al., 2021). Cronbach’s *α* in the current study was 0.84.

#### 2.3.4. Internalized homonegativity

IH was measured with the Revised Internalized Homonegativity Scale (IHS-R, Herek et al., 2009), Chilean version (Gómez et al., 2023). The IHS-R consists of five items rated on a 5-point scale (0 = never to 4 = often). Sample items include “If during the past year someone had offered you the opportunity to be completely heterosexual you would have accepted the offer” and “You have wished you were not gay/bisexual.” Higher scores indicate higher levels of IH. Previous studies provided evidence of adequate reliability, with Cronbach’s *α* from 0.79 (Huynh et al., 2020) to 0.82 (Herek et al., 2009). Reliability in this study was Cronbach’s *α* = 0.74.

### 2.4. Data analysis

Preliminary analyses included correlations between study variables at the individual and couple levels and testing of potential covariates to be included in the dyadic models. At the individual level, we computed correlations between different variables within partners (i.e., overall within-partner correlations). For couple-level correlations, we adopted a pairwise approach and computed intraclass correlations (ICCs) instead of standard interclass (Pearson) product–moment correlations (González and Griffin, 1997; Kenny et al., 2006), because partners in same-sex dyads are not distinguishable based on their sex and their designation as Partner 1 or Partner 2 is arbitrary. Following González and Griffin (1997), we computed pairwise ICCs for correlations between both partners’ reports of the same variables to test for interdependence within dyads, and cross-ICCs for correlations between different variables between partners. A *z*-statistic was computed to test for the statistical significance of correlations while adjusting for the interdependence between dyad members’ reports (González and Griffin, 1997). To test for the need to include covariates in the dyadic models, emotional intimacy and IH were correlated with relationship length and compared (ANOVA) across groups based on couple-level union, cohabitation, and parental status, education, and employment. Variables that were significantly associated with the outcome or moderator were included as covariates in the dyadic models.

To test for the dyadic relationships between attachment insecurity and emotional intimacy and the moderating role of actor and partner IH, we used the APIMoM for indistinguishable dyads with a mixed moderator which varies between and within dyads (Garcia et al., 2015). APIMoM analyses were conducted within a structural equation modeling (SEM) framework (Olsen and Kenny, 2006; Ledermann and Kenny, 2017), using maximum likelihood estimator. Two APIMOMs were estimated, one for each romantic attachment dimension. In addition to actor and partner main effects, four moderation effects were estimated and tested: (1) actor’s IH moderating the relationship between actor’s attachment insecurity and actor’s intimacy (i.e., actor-moderated actor effect); (2) partner’s IH moderating the relationship between actor’s attachment insecurity and actor’s intimacy (i.e., partner-moderated actor effect); (3) actor’s IH moderating the relationship between partner’s attachment insecurity and actor’s intimacy (i.e., actor-moderated partner effect); and (4) partner’s IH moderating the relationship between partner’s attachment insecurity and actor’s intimacy (i.e., partner-moderated partner effect). The moderation effects were obtained by creating interaction terms between the grand-mean centered predictor and the grand-mean centered moderator (Aiken and West, 1991). To test for the significance of the four moderation effects combined, a reduced model with no interaction terms was estimated and compared against the moderation model (Garcia et al., 2015). A significant *χ^2^* difference test (Δ*χ^2^*) would reflect a significant decrease in fit in the reduced model relative to the moderation model, indicating the presence of a combined moderation effect and the need to inspect the interaction effects. In case of nonsignificant interaction terms, the model was re-run including only the significant moderation effects for model parsimony. In case of a significant interaction effect, simple slopes analysis was conducted. In simple slope analysis, the relevant (actor or partner) simple effects of attachment insecurity on emotional intimacy were examined at low (1 *SD* below the mean) versus high (1 *SD* above the mean) levels of the (actor or partner) moderator (Preacher et al., 2006).

Because dyad members were indistinguishable, means, variances, intercepts, residual variances, and covariance matrices were constrained to be equal across partners, in addition to equal actor and partner effects (Olsen and Kenny, 2006; Peugh et al., 2013). Model fit was evaluated following the steps outlined by Peugh et al. (2013) to remove misfit due to arbitrary designation of dyad members as Partner 1 or Partner 2 (Woody and Sadler, 2005). We estimated null (i.e., all covariances fixed to zero), saturated (i.e., all covariances freely estimated), and analysis models (i.e., hypothesized associations freely estimated), and computed adjusted model fit indexes for the hypothesized analysis model. Model fit was considered acceptable if the *χ^2^* was nonsignificant, the root mean square error of approximation (RMSEA) was ≤0.08, and the comparative fit index (CFI) was ≥0.90 (Hu and Bentler, 1999).

Statistical significance was set at *p* < 0.05. For interpretation of effect size, ICCs and Pearson’s *r* of 0.10 were considered small, 0.30 medium, and 0.50 large (Cohen, 1988). APIMoMs and simple slope analyses were performed using Mplus 7.2, and all other analyses using IBM SPSS 27.

## 3. Results

### 3.1. Preliminary analyses

Results of preliminary analyses are presented in Table 1. Pairwise ICCs were significant for emotional intimacy and IH, which were both positively associated between partners, with small-to-medium effect size. Attachment anxiety and attachment avoidance did not correlate between partners, consistent with previous research (Campbell et al., 2001; Barry and Lawrence, 2013). As indicated by overall within-partner correlations and cross-ICCs, attachment anxiety and avoidance were positively associated within partners, with small effect sizes, and there was a small positive correlation between one partner’s attachment anxiety and the other partner’s attachment avoidance. Both attachment anxiety and attachment avoidance were negatively associated with emotional intimacy, with small-to-medium effect sizes. Correlations between attachment insecurity and IH were nonsignificant, except for a positive, small correlation between attachment avoidance and IH at the individual within-partner level. Emotional intimacy and IH were not significantly associated.

**Table 1:** Within- and between-partner correlations, covariate testing, and descriptive statistics.

	Attachment anxiety	Attachment avoidance	Emotional intimacy	IH
Attachment anxiety BP	**0.150**			
Attachment avoidance WP	0.232^***^			
Attachment avoidance BP	0.244^***^	**0.028**		
Emotional intimacy WP	−0.203^***^	−0.376^***^		
Emotional intimacy BP	−0.155^**^	−0.243^***^	**0.294** ^***^	
IH WP	0.113	0.119^*^	−0.120	
IH BP	0.025	0.109	−0.021	**0.243** ^**^
**Covariates**				
Relationship length			0.015	−0.102
Cohabitation status			0.28	0.03
Union status			0.93	0.07
Parental status			2.04	2.68
Education			0.18	0.37
Job			0.01	0.49
*M* (*SD*)	21.95 (8.48)	12.79 (6.36)	22.70 (2.74)	7.04 (3.27)

IH, internalized homonegativity; WP, within-partner correlations; BP, between-partner correlations. Pairwise ICCs are in bold. Statistical significance of pairwise ICCs, overall within-partner correlations and cross-ICCs was calculated using z scores (González and Griffin, 1997). F statistics are displayed for all covariates except relationship length, for which Pearson’s *r* is displayed. *p ≤ 0.05, **p ≤ 0.01, ***p ≤ 0.001.

None of the couple-level characteristics was significantly associated with emotional intimacy or IH. Therefore, no covariates were included in the APIMoMs.

### 3.2. The effect of attachment anxiety on emotional intimacy moderated by IH

For the dyadic model with attachment anxiety as the predictor, the reduced model showed no decrease in fit compared to the moderation model, Δ*χ^2^*(4) = 1.98, *p* = 0.74, indicating no moderation effects of IH. The final model, with the nonsignificant effects removed, showed adequate fit, *χ^2^*(2) = 3.26, *p* = 0.20, RMSEA = 0.07, CFI = 0.96. As displayed in Table 2, actor and partner associations between attachment anxiety and emotional intimacy were both significant and negative, indicating that higher levels of actor’s and partner’s attachment anxiety were associated with lower actor’s emotional intimacy.

**Table 2:** Effects in the final dyadic models.

	*b*	*SE*	*z*	95% CI
**Attachment anxiety**				
Actor’s anxiety	−0.18	0.06	−3.25^**^	[−0.30, −0.07]
Partner’s anxiety	−0.13	0.06	−2.23^*^	[−0.24, −0.02]
**Attachment avoidance**				
Actor’s avoidance	−0.37	0.05	−7.42^***^	[−0.46, −0.27]
Partner’s avoidance	−0.24	0.05	−4.71^***^	[−0.35, −0.14]
Partner’s avoidance × Actor’s IH	0.13	0.06	2.40^*^	[0.03, 0.24]
Partner’s avoidance × Partner’s IH	−0.14	0.05	−2.61^**^	[−0.25, −0.04]

b = standardized estimate; SE, standard error; CI, confidence interval; IH, internalized homonegativity. ^*^p < 0.05, ^**^p < 0.01, ^***^p < 0.001.

### 3.3. The effect of attachment avoidance on emotional intimacy moderated by IH

For the dyadic model with attachment avoidance as the predictor, the reduced model showed a poorer fit than the moderation model, Δ*χ^2^*(4) = 13.83, *p* = 0.008, indicating the presence of moderation effects. Inspection of interaction effects revealed significant actor-moderated, *b* = 0.16, *SE* = 0.06, *z* = 2.81, *p* = 0.005, 95% CI [0.05, 0.27], and partner-moderated, *b* = −0.16, *SE* = 0.06, *z* = −2.85, *p* = 0.004, 95% CI [−0.26, −0.05], partner effects. The nonsignificant actor-moderated, *b* = −0.04, *SE* = 0.06, *z* = −0.76, *p* = 0.45, 95% CI [−0.15, 0.07], and partner-moderated, *b* = 0.10, *SE* = 0.06, *z* = 1.81, *p* = 0.07, 95% CI [−0.01, 0.21], actor effects were removed for model parsimony and the APIMoM was re-run.

**Figure 1:**
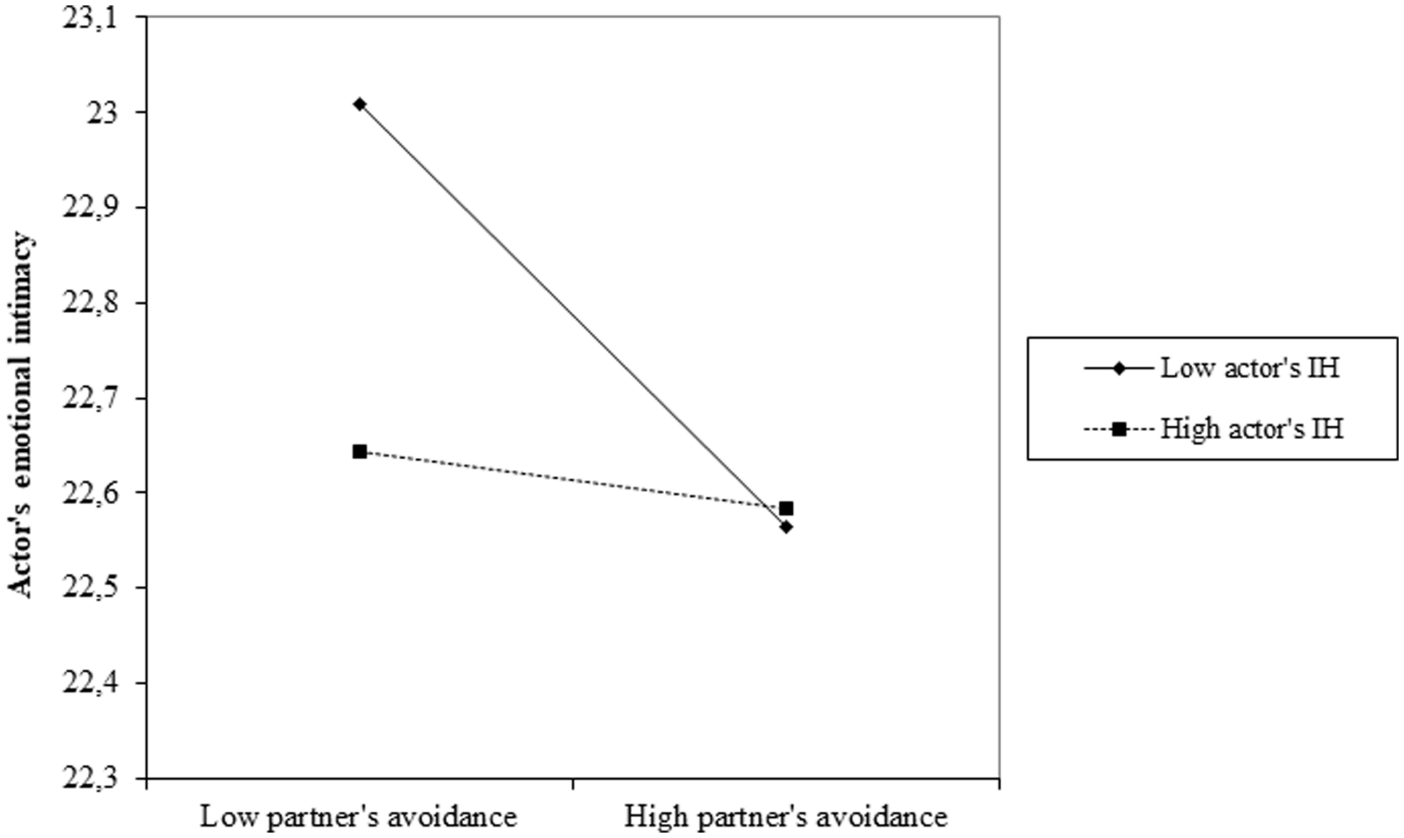
The association of partner’s attachment avoidance with actor’s emotional intimacy as a function of actor’s internalized homonegativity (IH).

The final model, with the nonsignificant effects removed, showed adequate fit to the data, *χ^2^*(2) = 3.37, *p* = 0.19, RMSEA = 0.07, CFI = 0.99. As reported in Table 2, actor and partner effects were both significant and negative, indicating that higher actor’s and partner’s attachment avoidance were both associated with lower actor’s emotional intimacy. As for the significant actor-moderated partner effect (Partner’s avoidance x Actor’s IH in Table 2), simple slope analysis showed that the negative associations of partner’s attachment avoidance with actor’s emotional intimacy was statistically significant at low, *b* = −0.19, *SE* = 0.04, *z* = −4.65, *p* < 0.001, 95% CI [−0.27, −0.11], but not high, *b* = −0.06, *SE* = 0.04, *z* = −1.77, *p* = 0.08, 95% CI [−0.13, 0.01], levels of actor’s IH (Figure 1).

**Figure 2:**
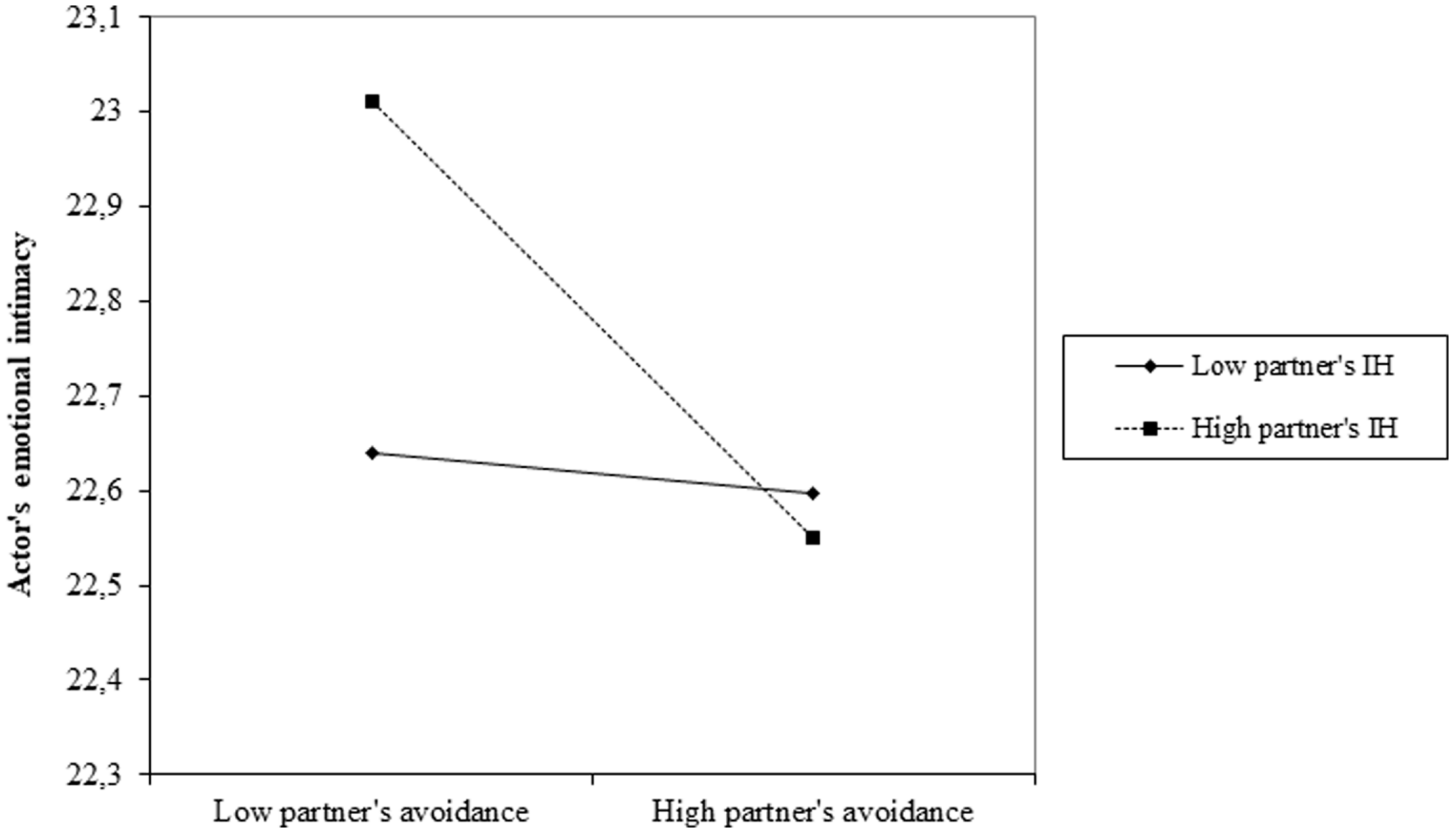
The association of partner’s attachment avoidance with actor’s emotional intimacy as a function of partner’s internalized homonegativity (IH).

As for the partner-moderated partner effect (Partner’s avoidance x Partner’s IH in Table 2), analysis of simple slopes revealed that the negative association of partner’s attachment avoidance with actor’s emotional intimacy was statistically significant at high, *b* = −0.20, *SE* = 0.04, *z* = −5.15, *p* = <0.001, 95% CI [−0.27, −0.12], but not low, *b* = −0.06, *SE* = 0.04, *z* = −1.45, *p* = 0.15, 95% CI [−0.13, 0.02], levels of partner’s IH (Figure 2).

## 4. Discussion

The present study aimed to fill a gap in the literature and examine whether IH moderated the association between attachment insecurities and emotional intimacy in same-sex male couples. By adopting a dyadic perspective, we examined the relationships of one’s own and partner’s attachment insecurities with one’s own emotional intimacy, and the potential role of each partner’s IH in moderating these dyadic associations.

Altogether, our findings reinforce the consideration of attachment theory as a conceptual framework that explains differences in the way closeness and intimacy are regulated within the couple relationship (Feeney, 2016; Mikulincer and Shaver, 2016). Indeed, as expected, attachment insecurity was linked to lower emotional intimacy at both actor and partner levels, in line with previous dyadic studies of heterosexual people (Dandurand and Lafontaine, 2013; Gagné et al., 2021). Moreover, coherent with previous evidence, attachment avoidance was more strongly related to lower emotional intimacy than attachment anxiety (Pielage et al., 2005; Constant et al., 2021; Gagné et al., 2021).

For both attachment orientations, actor-level associations were not moderated by IH. Thus, regardless of one’s own and the partner’s IH levels, the one’s own higher attachment anxiety and attachment avoidance were associated with one’s own lower perceptions of being intimate and experiencing closeness. According to attachment theory, more anxiously attached individuals usually have important needs for reassurance, love, connection, and crave for proximity, along with the perception of low responsiveness and care from their partners (Feeney and Noller, 1991; Gagné et al., 2021; van Lankveld et al., 2021). These characteristics, typically anchored in the chronic activation of the attachment needs (hyperactivation strategies), can interfere with the possibility of experiencing emotional closeness and a deep connection in the romantic bond (Mikulincer and Shaver, 2016). As for avoidant attachment, the tendency of avoidantly attached individuals to keep emotional distance from others, their need of autonomy, and their negative model of others, may hinder their willingness to seek closeness and to be involved in a depth communication (Feeney, 2016; van Lankveld et al., 2021), thus contributing to the lower perceived/reported emotional intimacy/closeness within their couple. These characteristics are based on the deactivating strategies of the attachment needs typical in individuals with higher avoidance attachment (Mikulincer and Shaver, 2016). Therefore, both forms of individual attachment insecurities represent vulnerability factors in the perception of intimacy within the couple (Gagné et al., 2021), independently of individual and partner IH.

At the partner level, the partner’s higher attachment anxiety was associated with one’s own lower emotional intimacy, regardless of one’s own and the partner’s IH levels. It is possible that anxiously attached individuals, due to intrusiveness in their behavior and exacerbated needs of closeness and proximity that are translated into pursuing and protest behaviors, facilitate a distance/withdraw response pattern in their partners (Collins and Read, 1990; Feeney and Noller, 1991; Bradford et al., 2002).

Contrary to our expectations, actor’s IH did not moderate the actor-level associations of attachment anxiety and attachment avoidance with emotional intimacy, and partner’s IH had no moderating role on the partner-level association between attachment anxiety, attachment avoidance, and emotional intimacy. Although these results can be counterintuitive, it can be hypothesized that, at the individual level, the nature of the associations between attachment, emotional intimacy, and IH is different, for example, through a mediational model, in which actor’s attachment insecurities are associated to higher levels of IH, which, in turn, decreases the perceived levels of emotional intimacy. Future studies, with appropriate designs (e.g., longitudinal studies), can elucidate this question. Another possible explanation in the case of attachment anxiety, is that the size of the effects of attachment anxiety on components of relationship functioning has been reported as low in previous research, and interaction effects are typically smaller than main effects (Blake and Gangestad, 2020). Therefore, it is possible that our study was underpowered to detect moderation effects for attachment anxiety. Further studies using larger samples are needed to clarify this issue.

We found significant actor- and partner-moderated partner effects for attachment avoidance. Thus, the association of the partner’s attachment avoidance with one’s own emotional intimacy was moderated by one’s own and the partner’s IH levels. Regarding the moderating role of individual IH, simple slope analysis revealed that only for individuals with low IH, their partner’s higher attachment avoidance was associated with their own lower emotional intimacy. This suggests that if a dyad member has low IH, his emotional intimacy will be negatively affected by his partner’s attachment avoidance. Being partnered with someone who has high attachment avoidance may especially frustrate one’s own connection needs, leading to lower feelings of validation, caring, and acceptance by the partner, when one’s own IH is low. Low IH entails more acceptance of the sexual orientation, less shame, and less efforts to conceal the relationship from others, along with more positive attitudes toward LGBT people. Under these conditions, the avoidance of the partner seems to directly challenge the intimacy needs and perceptions of those men who are low in IH, and thus possibly more invested in same-sex relationships. Conversely, under conditions of high individual IH, the partner’s attachment avoidance was unrelated to one’s own emotional intimacy. Therefore, a high individual IH seems to buffer the negative effects of the partner’s attachment avoidance on one’s own perceptions of closeness and sharing of feelings and experiences. It is therefore likely that for those having a high IH, probably expressed in less positive attitudes toward one’s own sexual orientation, the partner’s avoidant behaviors corroborate their own negative relational disposition, with no negative consequences on their feelings of intimate connection.

Regarding the moderating role of partner’s IH, results of simple slope analysis showed that, as hypothesized, higher partner’s attachment avoidance was linked to actor’s lower emotional intimacy only at high levels of partner’s IH. Therefore, being partnered with someone who has high IH seems to heighten the negative effects of the partner’s attachment avoidance on one’s own emotional intimacy. Individuals high in IH are likely to show reduced relational trust and withdrawal from the romantic relationship (Doyle and Molix, 2015; Mohr and Jackson, 2016). Therefore, a high IH in avoidantly attached individuals, who are more emotionally detached and reluctant to self-disclosure, might accentuate their deactivating strategies in intimate relationships and make their partners feeling less connected and having more unmet needs of understanding, support, and affirmation. The association of partner’s attachment avoidance with one’s own emotional intimacy was instead nonsignificant at low levels of the partner’s IH. Thus, for those men whose partners do not harbor negative views of their sexual orientation and express greater acceptance of their identity, their own perception of emotional connection and expectations of mutual caring is not affected by their partner’s attachment avoidance.

Hence, expanding previous research, we identified that a proximal minority stressor like IH moderates the partner association between attachment avoidance and emotional intimacy. Notably, results from our study highlight that the holder of IH (actor or partner) is key to its moderating role. Specifically, whereas the partner’s higher IH enhances the negative effects of the partner’s avoidance on the actor’s relational intimacy, the actor’s higher IH inhibits it. The moderating role of the partner’s IH was consistent with our hypothesis, but that of the actor’s IH was unexpected. This latter result opens interesting avenues to understand the interplay of partners’ IH and attachment avoidance in predicting emotional intimacy. For instance, it is possible that men who internalize homonegativity to a greater extent experience lower emotional intimacy altogether, and that this discomfort with their own sexual orientation makes the partner’s avoidance irrelevant to their perceived emotional intimacy—that is, a high internalization of homonegativity by men involved in same-sex relationships may limit their ability to be affected by their partner’s avoidance.

Our findings are in line with minority stress theory (Frost and Meyer, 2009), and expand previous research by demonstrating how one’s own and the partner’s levels of IH can reduce or enhance, respectively, the negative partner effects of attachment avoidance on emotional intimacy in same-sex male couples. In other words, IH of both dyad members moderates the effects of one partner’s attachment avoidance on the other partner’s emotional intimacy.

### 4.1. Limitations and future directions

Despite its contributions, the present study is not without limitations. First, the correlational design prevents from drawing any conclusion about causality. Longitudinal dyadic models of emotional intimacy over time should be tested to verify the temporal order of the associations of attachment insecurity and IH with emotional intimacy. However, assuming attachment as predictor of emotional intimacy and IH as a moderator is coherent with the notion that attachment insecurities represent an individual vulnerability whose effects can be enhanced in presence of stressors such as IH. Second, we exclusively considered self-reported attachment insecurity, IH, and emotional intimacy. Future research using both self- and partner-reports would deepen our understanding of the interplay between attachment insecurity and IH for the couple’s functioning, besides reducing common method variance (Orth, 2013). Also, to assess IH, we used the Revised Internalized Homonegativity Scale, which provides a global IH score, because it has been validated for use with Chilean LGB individuals (Gómez et al., 2023). However, it would be interesting that future studies use other measures that consider different components of IH, such as public identification as a sexual minority and sexual and social comfort with sexual minority individuals (Currie et al., 2004; Morell-Mengual et al., 2017). This would enhance our understanding of how sexual minority stressors affect couple relationship dynamics, by elucidating whether and how different components of IH differently moderate the relationship between romantic attachment and emotional intimacy in same-sex male couples. Third, the great majority of couples in our sample were relatively recent (having been together for less than 5 years) and only a few couples were in a civil union, which may limit the generalizability of our findings. However, it is worth noting that in Chile a law allowing civil union between same-sex partners was only passed in 2015 (Biblioteca del Congreso Nacional de Chile, 2015), and that same-sex marriage was approved in 2021. Therefore, it would be interesting to investigate whether our findings are replicated in culturally and demographically diverse samples, such as long-term or married same-sex male couples. Fourth, we only included same-sex male couples in the current study. Thus, replication studies including other LGBT couples would be important to examine whether the same pattern of associations holds among other kind of LGBT couples. This would also be especially valuable in providing that all the groups that constitute the LGBTQ+ acronym are considered in the research domain, thus preventing that individuals from sexual minority identities other than gay and lesbian live a condition of double-invisibility (Salvati and Koc, 2022). Finally, we focused on the moderating role of IH, but other components of minority stress (i.e., discrimination experiences at the couple level, sexual orientation concealment) as well as relational variables, such as dyadic coping, might intervene to influence the dyadic associations between attachment insecurity and emotional intimacy among same-sex couples.

### 4.2. Implications and conclusions

The present study was the first study to investigate the dyadic interactive effects of attachment insecurity and IH on emotional intimacy among male same-sex couples. Our findings add to previous consistent evidence of the role of attachment insecurity for relationship functioning, by showing that the same negative effects are observed in same-sex couples. Moreover, this was the first dyadic study conducted in a Latin American sample, which expands current knowledge to a culturally diverse sample. This is especially relevant as couple-based studies in the LGBT population are scarce in Latin America and Chile, with some exceptions (Guzmán-González et al., 2020b).

As for the clinical implications, our results revealed that IH constitutes a risk factor whose effect needs to be addressed when working with couples, providing insights of the importance of considering the specific needs and challenges faced by same-sex couples when designing couple interventions. Our empirical results, if replicated in more diverse LGBT couples, highlight the relevance of considering minority stressors for each partner. Thus, helping couples to recognize and handle the influence of minority stressors on their relationship might promote intimacy, an approach that may be especially relevant when one of the partners is more avoidantly attached.

Taking together, our results reveal that the integration of two theoretical frameworks, such as attachment theory and minority stress theory, represents a potentially fertile avenue for future research on LGBT couple functioning.

## Data availability statement

The datasets analyzed during the current study are not publicly available due to privacy issues related to the participants, but are available from the corresponding author by reasonable request.

## Ethics statement

The studies involving human participants were reviewed and approved by Comité Etico Científico Universidad Católica del Norte (CEC-UCN). The patients/participants provided their written informed consent to participate in this study.

## Author contributions

MG-G: funding acquisition, project administration, conceptualization, methodology, writing-original draft preparation, and writing-review and editing. GC: methodology, formal analysis, interpretation of results, and writing-review and editing. FG: writing and editing. JoB, JaB, LG-R, and RE-T: writing-review and editing. All authors contributed to the article and approved the submitted version.

## Funding

This research was funded by the National Fund for Scientific and Technologic Development of Chile, FONDECYT 1190240.

## Conflict of interest

The authors declare that the research was conducted in the absence of any commercial or financial relationships that could be construed as a potential conflict of interest.

## Publisher’s note

All claims expressed in this article are solely those of the authors and do not necessarily represent those of their affiliated organizations, or those of the publisher, the editors and the reviewers. Any product that may be evaluated in this article, or claim that may be made by its manufacturer, is not guaranteed or endorsed by the publisher.
